# Prenatal diagnosis and outcomes in 320 fetuses with nasal bone anomalies

**DOI:** 10.3389/fgene.2023.1170720

**Published:** 2023-08-24

**Authors:** Hui Li, Yanyi Yao, Chengcheng Zhang, Yayun Qin, Ling Zeng, Jieping Song, Li Lu, Wei Wang, Lijun Liu

**Affiliations:** ^1^ Medical Genetic Center, Maternal and Child Health Hospital of Hubei Province, Tongji Medical College, Huazhong University of Science and Technology, Wuhan, China; ^2^ Department of Ultrasonography, Maternal and Child Health Hospital of Hubei Province, Tongji Medical College, Huazhong University of Science and Technology, Wuhan, China; ^3^ Department of Pediatric Respiratory, Maternal and Child Health Hospital of Hubei Province, Tongji Medical College, Huazhong University of Science and Technology, Wuhan, China

**Keywords:** prenatal diagnosis, absent nasal bone, hypoplastic nasal bone, aneuploidy, copy number variations

## Abstract

**Object:** To investigate the chromosome abnormalities associated with absent or hypoplastic fetal nasal bone.

**Methods:** Patients with fetal nasal bone anomalies (NBA) referred to our center for prenatal diagnosis between 2017 and 2021 were retrospectively evaluated. All these patients underwent chromosomal microarray and/or karyotyping and received genetic counseling before and after testing.

**Results:** Among 320 fetuses with NBA, chromosomal abnormalities were diagnosed in 89 (27.8%) cases, including 53 cases of trisomy 21, which was the most common type of chromosomal aneuploidy, accounting for 59.6% of all detected abnormalities. In addition to aneuploidies, 29 cases of copy number variants (CNVs) were detected. In cases of isolated NBA with low-risk screening results and without other risk factors, the incidence of fetal chromosomal aneuploidies and pathogenic CNVs is 5.3% (7 in 132 cases).

**Conclusion:** This study suggests that parents of fetuses should be informed about the possibility of fetal aneuploidy and pathogenic CNVs and that discussion with the parents is also recommended, providing data support and reference for clinical counseling.

## 1 Introduction

During early human embryonic development, nasal bone (NB) develop in the membrane of the dense mesenchyme overlying the cartilaginous nasal capsule. They first become visible histologically at 9–10 weeks, and have been demonstrated by ultrasound as a white line behind the fetal skin (Miller et al., 2023). The absence of nasal bones is currently considered to be one of the highly specific soft markers for fetal chromosomal abnormalities, regardless of gestational age (Prabhu et al., 2021). The most common chromosomal abnormality associated with nasal bone anomalies is trisomy 21, but others such as trisomy 18, trisomy 13, sex chromosomal abnormalities, and other rare abnormalities have also been reported (Moczulska et al., 2022; Shi et al., 2022). Current studies agree that invasive prenatal diagnosis should be performed for fetal nasal abnormalities in combination with other sonographic abnormalities (Lostchuck and Hui, 2019). However, there is disagreement among different researchers as to how prenatal genetic counseling should be performed for isolated NBA. Fetal nasal bone length in the second trimester is inconsistent among different ethnic groups, and that ethnic differences may affect nasal bone performance during pregnancy, which may affect screening performance (Papasozomenou et al., 2016). Fantasia suggested that the risk of pathogenic CNVs in fetuses with nasal bone abnormalities in early pregnancy is mainly related to the thickened NT rather than the absence of nasal bone itself (Fantasia et al., 2020). Chen believed that nasal bone is an important marker of chromosomal abnormalities in some populations, but less so in the Chinese Han population. In most cases of early nasal bone hypoplasia, which may be caused by delayed ossification of NB, NB can be seen on the second scan of the second trimester. Non-invasive prenatal screening (NIPS) is the first choice of follow-up for fetal NB dysplasia in the first trimester (Chen et al., 2020). Gu suggested that NB dysplasia should be considered not only as a marker for trisomy 21, but also as an objective marker for facial malformations associated with clinically relevant copy number variants (CNVs). Regardless of whether routine screening for chromosomal aneuploidy is low-risk or not, if ultrasound suggests fetal nasal dysplasia, tertiary ultrasound should be performed and invasive diagnosis with chromosomal microarray analysis (CMA) should be considered (Gu et al., 2019a; b).

This study retrospectively analyzed the data of pregnant women who came to our prenatal diagnosis center for consultation and received invasive prenatal diagnosis of absent or hypoplastic fetal nasal bone from 2017 to 2021, and analyzed the detection of fetal chromosomal abnormalities and prognosis, to provide a database for clinical consultation.

## 2 Materials and methods

### 2.1 Patient population

From May 2017 to December 2021, cases with absent or hypoplastic fetal nasal bone identified by ultrasound, whose parents consented and underwent an invasive prenatal diagnosis by CMA and/or karyotyping were enrolled and clinical data and laboratory results were analyzed.

### 2.2 Ultrasonographic diagnostic criteria

Nasal bone can be seen in a mid-sagittal view of the fetal profile during the first trimester of pregnancy. Absent or hypoplasia of the nasal bone is a single white line representing fetal skin in the fetal nasal region, but no clean second line of the nose on the monitor.

### 2.3 Invasive procedure and fetal chromosome karyotyping

The high-risk pregnant women underwent amniocentesis after genetic counseling. Routine amniocentesis was performed between 17 and 36 weeks of gestation under ultrasound guidance by two experienced physicians, and 20 mL of amniotic fluid was collected for laboratory analysis. Fetal chromosome karyotyping was performed in pregnant women under 26 weeks of gestation on the cultured cells from trophoblastic cells in amniotic fluid using a G-binding method.

### 2.4 Chromosome microarray analysis

Amniotic fluid genomic DNA was extracted using the QIAamp DNA mini kit (Qiagen). CMA was performed using the Cytoscan 750K array (including 550,000 CNV probes and 200,000 SNP probes) according to the manufacturer’s instructions (Affymetrix, Santa Clara, CA, United States). Data analysis was performed using Affymetrix Chromosome Analysis Suite software version 3.3. Reference databases included Online Mendelian Inheritance in Man (OMIM, http://www.ncbi.nlm.nih.gov/omim), Database of Genomic Variation and Phenotype in Humans Using Ensembl Resources (DECIPHER, http://decipher.sanger.ac.uk/), ClinGen Dosage Sensitivity Map (ClinGen, https://www.ncbi.nlm.nih.gov/projects/dbvar/clingen/), and others. According to the American College of Medical Genetics (ACMG) practice guidelines, CNVs have been classified into 5 categories: pathogenic (P), likely pathogenic (LP), variants of uncertain significance (VOUS), likely benign and benign (Riggs et al., 2020). Only the first three types of CNVs have been reported.

### 2.5 Follow-up

Clinical information and pregnancy outcomes were collected from referring physicians and medical records. Additional follow-up information was obtained by telephone interview including delivery, postnatal growth and physical examination, major malformations and other complications.

### 2.6 Statistical analysis

Statistical analysis was performed with chi-squared in relation to different parameters, and *p* < 0.05 was considered statistically significant. All analyses were performed with SPSS 22.0 version.

## 3 Results

During the study period, all the 320 fetuses with prenatally diagnosed absent or hypoplastic NB received an invasive prenatal diagnosis by CMA testing while 228 cases also underwent karyotyping within 26 weeks of gestation. The overall detection rate of fetal chromosomal abnormalities was 27.81% (89/320).

Maternal age ranged from 16 to 43 years, 86.88% (278/320) were younger than 35 years. A significant difference was observed in the detection rate of fetal chromosomal abnormality between pregnant women of advanced maternal age (AMA) (≥35 years) and normal age (<35 years) (45.24% vs 25.18%, χ^2^ = 7.312, *p* < 0.05). Thirty-one fetuses (9.69%, 31/320) were diagnosed by ultrasound in the first trimester, and 289 (90.31%, 289/320) diagnosed in the second or third trimester. Chromosomal abnormalities were detected in 61.29% (19/31) of cases with NBA in the first trimester, while 24.22% (70/289) in cases in the second or third trimester and the difference was statistically significant (χ^2^ = 19.161, *p* < 0.001) (Table 1).

**TABLE 1:** Maternal and pregnancy characteristics (*n* = 320).

Category	Genetic results (*n*)	
	Abnormal	Normal
Maternal age (years)	30 (22–41)	28 (16–43)
Median (Range)		
Maternal age ≥35 y	19	23
Maternal age <35 y	70	208
Gestational age (weeks)^a^	23 (17–36)	25 (17–33)
Median (Range)		
First-Trimester^b^	19	12
Second-/Third-Trimester^b^	70	219

^a^ Gestational age of amniocentesis.

^b^ Gestational age of first discovery of fetal nasal bone abnormality.

Chromosomal abnormalities were diagnosed in 89 cases, including 53 cases of trisomy 21, 4 cases of trisomy 18, 1 case of mosaic trisomy 13, 4 cases of sex chromosome aneuploidies, and 1 case of mosaic trisomy 2. Except for this case of mosaic trisomy 2, CMA identified all aneuploidies detected by karyotyping and an additional 29 cases of CNVs (including 2 cases combined with trisomy 21 and 1 case combined with mosaic 45,X) (Tables 2–4;). All cases with trisomy 21, trisomy 18, and trisomy 13 opted for pregnancy termination. In the case of mosaic trisomy 2, the mother chose to continue the pregnancy and gave birth to a girl who was followed up for 2 years and no abnormality was found.

**TABLE 2:** Summary of chromosomal aberrations among the 320 fetuses with nasal bone abnormality.

Genetic results	First-trimester		Second-/Third trimester	
	Isolated	Non-isolated	Isolated	Non-isolated
Aneuploidies				
T21	0	12	7	34
T18	0	1	0	3
T13	0	0	1^a^	0
XXY	1	1	0	0
XO	0	1	1^a^	0
T2	0	0	1^a^	0
CNVs				
P	0	1	6^b^	8^d^
LP	1	1	2	4
VOUS	0	0	6^c^	1
Normal	8	4	156	63
Total	10	21	178	111

CNVs, copy number variations; P, pathogenic; LP, likely pathogenic; VOUS, variants of unknown/uncertain significance.

^a^ Mosaic.

^b^ Including 1 case of T21 combined with X-linked DMD carrier and 1 case of X-linked STS carrier.

^c^ Including 1 case of mosaic XO combined with VOUS-CNV.

^d^ Including 1 case of T21 combined with P-CNV and 1 case contained two P-CNVs.

**TABLE 3:** Summary of the cases with nasal bone abnormality identified with pathogenic/likely pathogenic CNVs by genetic amiocentesis (*n* = 19).

Case	MA (y)	GA (w)	Other ultrasonic findings	Aneuploidy	Copy number variations	Classifi-cation	Outcomes
				Screening			
1	28	27	None	cfDNA: LR	arr [GRCh37] 16p13.3 (3694760_4299989)x3	P	TOP
2	27	26	echoless midline brain, flat facial shape, micrognathia	cfDNA: LR	arr [GRCh37] 4p16.3p15.1 (68346_33755417)x1	P	TOP
			thoracolumbar scoliosis, abnormal left foot posture and SUA				
3	30	27	SUA	MSS: LR	arr [CRCh37] 11q13.4q25 (72744004_134937416)x3	P	TOP
4	26	23	SUA	cfDNA: LR	arr [CRCh37] 1q44 (243943235_249224684)x1	P	TOP
5	29	23	TCD < −3SD, SUA	cfDNA: LR	arr [GRCh37] 5p15.33p14.1 (113576_25370521)x1	P	TOP
					arr [GRCh37] 9p24.3p21.3 (208454_20653331)x3	P	
6	31	17	reversed a-wave in fetal ductus venosus	None	arr [GRCh37] 22q13.1q13.33 (38789935_51197766)x3	P	TOP
7	29	29	PLSVC	MSS: HR	arr [GRCh37] 19p13.2 (9736153_12602337)x1	P	TOP
8	27	23	None	cfDNA: LR	arr [GRCh37] 13q13.2q34 (35235990_115107733)x1-2	P	NLB, follow-up to 8 months showed no abnormality
9	29	27	None	cfDNA: LR	arr [GRCh37] 9p24.3p13.1 (208454_38772005)x3	P	TOP
10	29	24	bilateral superior vena cava, ductus venosus agenesis, enlarged inferior vena cava, enlarged right heart, pericardial effusion	MSS: LR	arr [GRCh37] 22q11.21 (18648856_21800471)x1	P	TOP
11	36	18	None	cfDNA: LR	arr [GRCh37] 9q31.2q33.2 (110873766_125177344)x1	P	TOP
12	32	18	NT 4.8 mm	None	arr [GRCh37] 16p11.2 (29428532_30176508)x1	LP	TOP
13	32	18	None	None	arr [GRCh37] 16p11.2 (28748617_29051191)x1	LP	TOP
14	30	27	polyhydramnios	cfDNA: LR	arr [GRCh37] 16p11.2 (29428532_30178406)x3	LP	NLB, follow-up to 24 months showed no abnormality
15	29	25	echogenic intracardiac focus, left aortic arch, aberrant right subclavian artery	MSS: LR	arr [CRCh37] 1q44 (224817841_246711631)x1	LP	Postnatal death
16	32	19	thickened NF, cystic hygroma of the neck, VSD	None	arr [CRCh37] 10q24.31q24.32 (102979827_103370322)x3	LP	TOP
17	38	26	polyhydramnios	cfDNA: LR	arr [GRCh37] 17p12 (14060292_15484335)x1	LP	NLB, follow-up to 7 months showed no abnormality
18	30	26	None	cfDNA: LR	arr [GRCh37] 17p12 (14060292_15513053)x1	LP	TOP
19	30	17	None	None	arr [CRCh37]1q21.1q21.2 (145390102_147844778)x3	LP	NLB, follow-up to 9 months with asymptomatic ASD

MA, maternal age; GA, gestational age; MSS, maternal serological screening; NT, nuchal translucency; cfDNA, cell-free DNA; LR, low risk; HR, high risk; SUA, single umbilical artery; PLSVC, permanent left superior vena cava; TCD, transverse cerebellum diameter; NF, nuchal fold; VSD, ventricular septal defect; ASD, atrial septal defect; ARSA, aberrant right subclavian artery; P, pathogenic; LP, likely pathogenic; TOP, termination of pregnancy; NLB, normal life birth.

**TABLE 4:** Cases with nasal bone abnormality identified with VOUS-CNVs by genetic amiocentesis (*n* = 7).

Case	MA (y)	GA (w)	Other ultrasonic findings	Aneuploidy	Copy number variations	Classifi-cation	Outcomes
				Screening			
20	27	23	None	MSS: LR	arr [GRCh37] 20p13 (61662_384403)x1	VOUS	TOP
21	26	24	cerebellar vermis dysplasia	MSS: LR	arr [CRCh37] 17p12q12 (14581083_34449108)x2 hmz	VOUS	TOP
22	30	24	None	MSS: LR	arr [CRCh37] 13q14.11q22.1 (41472024_75238097)x2 hmz	VOUS	NLB, follow-up to 18 months showed no abnormality
23	33	25	None	MSS: LR	arr [CRCh37] 17q25.3 (75631701_77439807)x3	VOUS	NLB, follow-up to 30 months showed no abnormality
24	25	24	None	cfDNA: LR	arr [GRCh37] 17q21.32 (45272307_45490519)x1	VOUS	NLB, follow-up to 20 months showed no abnormality
25	29	23	None	MSS: LR	arr [GRCh37] 14q21.3q22.1 (50752506_52184984)x3	VOUS	NLB, follow-up to 20 months showed no abnormality
26	33	24	None	cfDNA: LR	arr [GRCh37] Xp22.31 (6449752_8143509)x1	P (STS carrier)	NLB, follow-up to 20 months showed no abnormality

VOUS, variants of unknown/uncertain significance; P, pathogenic; TOP, termination of pregnancy; NLB, normal life birth.

Eleven (4.78%, 11/230) pathogenic CNVs ranging in size from 965 Kb to 79.9 Mb, including 1q44 deletion, 4p16.3p15.1 deletion, 5p15.33p14.1 deletion, 9q31.2q33.2 deletion, mosaic 13q13.2q34 deletion, 19p13.2 deletion, 22q11.21 deletion, 9p24.3p13.1 duplication, 9p24.3p21.3 duplication, 11q13.4q25 duplication, 16p13.3 duplication and 22q13.1q13.33 duplication. The fetus in case 8 had a low-level of mosaic 13q deletion. After extensive counseling, the parents decided to continue the pregnancy and had a normal live birth. The infant was followed up to 8 months of age and no significant abnormalities were observed. The other 10 cases with pathogenic CNVs decided to terminate the pregnancy (Table 3). Among the 8 cases (3.47%, 8/230) with likely pathogenic CNVs, 4 cases terminated pregnancy, 1 case the infant died after birth, 1 case was followed up to 9 months with asymptomatic atrial septal defect, and 2 cases were followed up to 7 months and 2 years, respectively, and no obvious abnormalities were found (Table 3). There are 6 cases with VOUS CNVs and 1 case with pathogenic X-linked CNVs, 2 of them chose to terminate pregnancy, and the others were followed up at least 18 months after birth and showed no obvious abnormalities (Table 4).

Isolated nasal bone anomalies (iNBA) occurred in 188 cases and non-isolated nasal bone anomalies (niNBA) in 132 cases. The fetuses with niNBA had a higher detection rate of chromosomal abnormalities than those with iNBA (49.24% vs. 12.77%, χ^2^ = 51.393, *p* < 0.001) (Table 2).

**FIGURE 1:**
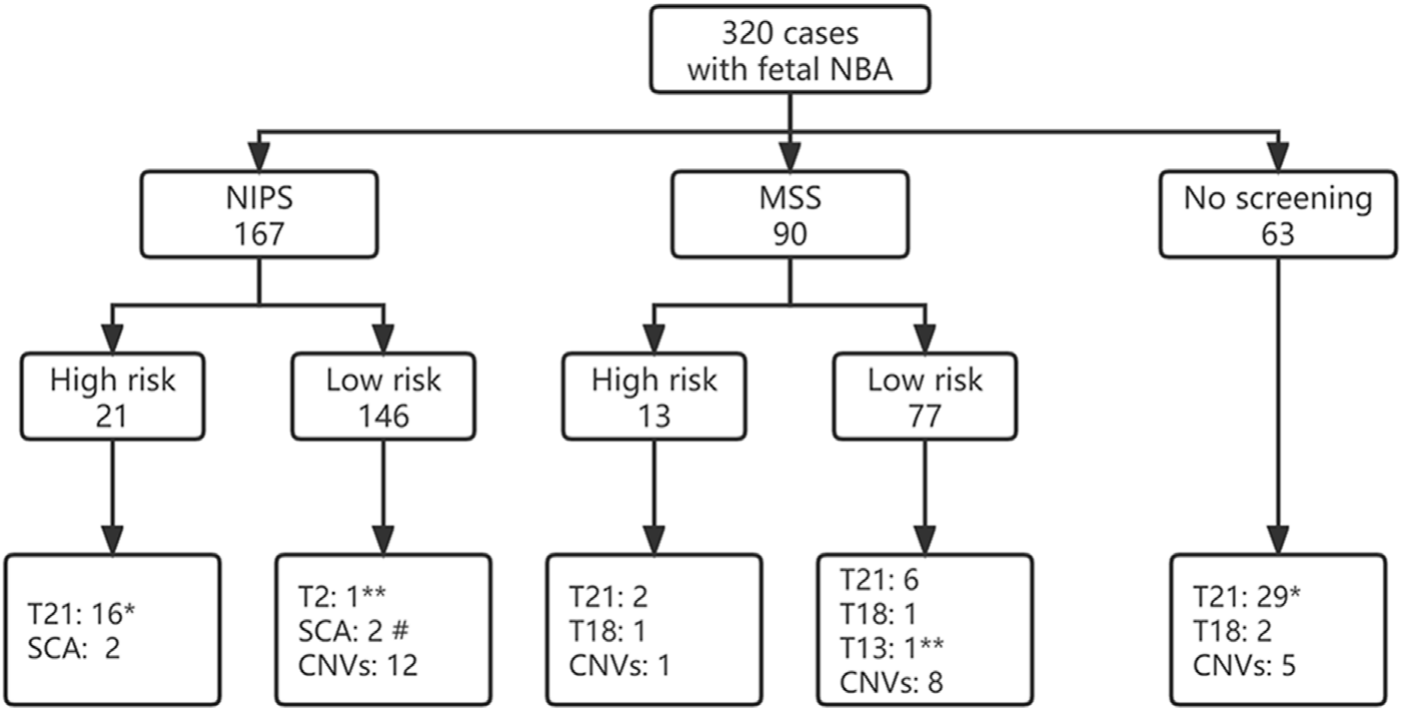
Fetal screening for nasal bone abnormalities and detection of fetal chromosome abnormalities. * including 1 case combine with CNV; # including 1 case of mosaic XO combined with CNV; ** mosaic. NBA, nasal bone anomalies; NIPS, non-invasive prenatal screening; MSS, maternal serological screening; CNVs, copy number variations.

Of 320 pregnant women, 167 cases received NIPS, 90 cases underwent maternal serologic screening (MSS), and 63 cases did not receive the above two screenings. Among the 223 cases with low risk of MSS or NIPS screening, chromosomal abnormalities were detected in 31 cases with a detection rate of 13.90%, while the chromosomal abnormalites rate was significantly higher in pregnant women with a high risk of screening (64.71%, 22/34), and the difference was statistically significant (χ^2^ = 46.517, *p* < 0.001). Among 63 cases without MSS or NIPS screening, 57.14% (36/63) of them were diagnosed with chromosomal abnormalities (Figure 1). Among them, 46 cases had directly received prenatal diagnosis due to nasal bone abnormalities combined with other high risk factors such as advanced maternal age, thickened NT, other ultrasound structural abnormalities or soft marker indicators, and so on, the detection rate was 69.57% (32/46), including 27 cases of T21 (one combined with CNVs), 2 cases of T18 and 3 cases of CNVs. In the other 17 cases of nasal bone abnormalities without any other high risk factors, 4 cases (23.53%, 4/17) were detected, including 2 cases of T21 and 2 cases of CNVs.

There were 132 cases of isolated fetal NBA with low-risk NIPS or MSS screening results and without other high-risk factors such as AMA, adverse pregnancy history, adverse environmental exposure, etc. The incidence of fetal chromosomal abnormalities was 5.3% (7/132), including 1 case of T21, 1 case of mosaic T2, 1 case of mosaic T13, 3 cases of P-CNVs and 1 case of LP-CNVs (case 1,8,9,18). In addition, 5 cases of VOUS-CNVs (cases 20, 22–25) and 1 case of pathogenic X-linked CNVs (case 26) were detected.

## 4 Discussion

Absence or hypoplasticity of NB has been considered as one of the strongest ultrasound markers for Down’s syndrome and other chromosomal abnormalities in the first and second trimester in previous studies (Canda et al., 2017; Du Y et al., 2018; Bernardeco et al., 2022). In our present study, 27.81% (89/230) of fetuses with NBA were found to have chromosomal abnormalities, including 53 cases of trisomy 21, which was the most common type of chromosomal aneuploidy and accounted for 59.55% (53/89) of all abnormalities detected. The proportion of all aneuploidies was 70.79% (63/89). This finding further supports the notion that absent or hypoplastic fetal nasal bone is an important marker for Down’s syndrome and other chromosomal abnormalities.

However, the relationship between the NBA and chromosomal microdeletions or microduplications is not well understood. CMA is a high-resolution molecular diagnostic technique that can effectively detect submicroscopic abnormalities such as chromosomal microdeletions and microduplications that cannot be detected by traditional karyotyping analysis (Liu et al., 2022). In the prenatal setting, with multiple advantages, CMA is the standard practice due to its higher resolution over conventional karyotyping and the ability of homozygosity and uniparental disomy for evaluation of fetuses with structural abnormalities or with soft markers (Xia et al., 2020; Hu et al., 2021). Huang et al. (2021) reported that the prevalence of chromosomal abnormalities was 25% (21/84) in fetuses with nasal bone hypoplasia in the second or third trimester, and CMA detected 10 more fetuses with CNVs, which increase the detection of genomic abnormalities by 11.9% as compared to G-banding karyotyping analysis, but no significant difference between the isolated nasal bone hypoplasia group and the non-isolated group. However, our data suggested that fetuses with non-isolated NBA had a significantly higher rate of chromosomal abnormalities than the isolated NBA group, which is consistent with Du’s study (Du Y et al., 2018). The difference may be related to the difference in sample size or gestational age of the fetal NBA included in the study.

The use of CMA in isolated fetal NBA is controversial. Lostchuck’s study showed that in 80 cases of isolated hypoplastic NB, eight (10%) of them had trisomy 21, but no pathogenic CNV or atypical chromosomal abnormality was found (Lostchuck and Hui, 2019). Chen believes that isolated NBA for trisomy 21 screening is less efficient in the Chinese population, and NIPS should be recommended in pregnant women aged ≥35 years in cases of absent or hypoplastic fetal nasal bone (Chen et al., 2020). Shi et al. (2022) found that the diagnostic yield of CMA for p-CNVs in fetuses with isolated absent or hypoplastic NB was 3.2%. Zhang et al. (2021) reported the fetal chromosomal abnormality rate of 5.71% and 15.0% in isolated fetal nasal bone absence or hypoplasia, respectively. Our data show that the detection rate of chromosomal abnormalities is 5.3% in cases of fetal iNBA with low risk of NIPS and/or MSS without other risk factors, and 9.85% when VOUS-CNVs was calculated, which is consistent with Gu’s report (Gu et al., 2019a). In our data, T21,T18, and T13 were not detected in the low-risk of NIPS group, while 1 case of T21 and 1 case of mosaic T13 were missed in the low-risk MSS group, showing that MSS had a lower efficiency than NIPS for common trisomies. Even in low-risk screening, there is still a possibility of a false negative (Evans et al., 2021). However, the performance of NIPS and NIPS-PLUS to detect CNVs is still limited, for different size of CNVs, the positive predictive value is inconsistent (Liang et al., 2019; Yang et al., 2021; Xue et al., 2022). In addition, our study was limited to the chromosomal level and did not include whole exome sequencing, so some of the pathogenic gene variants may be underestimated (Zhang et al., 2021; Moczulska et al., 2022).

Nasal bone development varies among individuals and ethnics groups (Chiu et al., 2011; Papasozomenou et al., 2016). Some nasal bone absence or hypoplasia is caused by internal or external factors in the embryonic development process, which leads to the interference of ossification process and delay of ossification. As a result, the nasal bone could not be observed by ultrasound in the early trimester, but could be recognized by ultrasound in the later stage (Miller et al., 2023). If the nasal bone can be measured in the second trimester, the vast majority of fetuses have a good prognosis. Our data showed that during the first trimester, most (80%, 8/10) fetuses with iNBA had normal chromosomal karyotypes. Isolated nasal bone absence or dysplasia in the first trimester can be followed up, and most nasal bones can be detected in the second trimester. Non-isolated nasal bone dysplasia at any gestational age usually predicts a more adverse pregnancy outcome.

Finally, from the patient’s point of view, they want to rule out as many abnormalities as possible to ensure the healthy condition of the fetus, taking into account social factors and reproductive costs. From the physician’s point of view, patients should be fully informed and consulted according to their condition. Therefore, during prenatal genetic counseling, physicians should also inform parents about the possibility of P-CNVs if the fetus has isolated NBA and discuss with them the benefits and risks of further invasive CNVs testing. The final decision for invasive prenatal diagnosis and CNVs testing remains the prerogative of the patient.

## 5 Conclusion

In conclusion, our data suggest that trisomy 21 was the most common chromosomal abnormality in fetuses with nasal bone anomalies, followed by trisomy 18 and sex chromosomal aneuploidy. And even with a low-risk routine aneuploidy screening result, performing a prenatal diagnosis of isolated nasal bone absence and hypoplasia, especially during the second and/or third trimester, increases the positive finding of fetal pathogenic CNVs or atypical chromosomal abnormalities. The possibility of the fetus carrying pathogenic CNVs shall be discussed with parents, and prenatal diagnostic CNVs testing should be recommended to avoid missed diagnosis. Our report adds to the published literature on the absence or dysplasia of fetal nasal bone. To provide data support and reference for clinical prenatal consultation.

## Data Availability

The data presented in this study are available on request from the corresponding author. The data are not publicly available due to the inclusion of protected health information. Requests to access these datasets should be directed to Lijun Liu, chrismcgrady@126.com.

## Abbreviations

NB, nasal bone; NBA, nasal bone anomalies; CNVs, copy number variants; NIPS, Non-invasive prenatal screening; CMA, chromosomal microarray analysis; P, pathogenic; LP, likely pathogenic; VOUS, variants of uncertain significance; AMA, advanced maternal age; iNBA, isolated nasal bone anomalies; niNBA, non-isolated nasal bone anomalies; MSS, maternal serologic screening.
